# Wilms Tumor in Sub-Saharan Africa: Molecular and Social Determinants of a Global Pediatric Health Disparity

**DOI:** 10.3389/fonc.2020.606380

**Published:** 2020-12-04

**Authors:** Annie Apple, Harold N. Lovvorn

**Affiliations:** ^1^ Vanderbilt University School of Medicine, Nashville, TN, United States; ^2^ Department of Pediatric Surgery, Monroe Carrell Jr. Children’s Hospital, Vanderbilt University Medical Center, Nashville, TN, United States

**Keywords:** Wilms tumor, sub-Saharan Africa, health disparity, molecular features, social determinants of health

## Abstract

Wilms tumor (WT) is the most common renal malignancy of childhood. Global disparities in WT have been reported with the highest incidence and lowest overall survival occurring in sub-Saharan African nations. After a detailed search of PubMed, we reviewed available literature on WT in sub-Saharan Africa and summarized findings that explore biologic and social factors contributing to this alarming cancer health disparity. Access to care and treatment abandonment are the most frequently reported factors associated with decreased outcomes. Implementation of multidisciplinary teams, collaborative networks, and financial support has improved overall survival in some nations. However, treatment abandonment remains a challenge. In high-income countries globally, WT therapy now is risk-stratified according to biology and histology. To a significantly lesser extent, biologic features have been studied only recently in sub-Saharan African WT, yet unique molecular and genetic signatures, including congenital anomaly-associated syndromes and biomarkers associated with treatment-resistance and poor prognosis have been identified. Together, challenges with access to and delivery of health care in addition to adverse biologic features likely contribute to increased burden of disease in sub-Saharan African children having WT. Publications on biologic features of WT that inform treatment stratification and personalized therapy in resource-limited regions of sub-Saharan Africa have lagged in comparison to publications that discuss social determinants of health. Further efforts to understand both WT biology and social factors relevant to appropriate treatment delivery should be prioritized in order to reduce health disparities for children residing in resource-limited areas of sub-Saharan Africa battling this lethal childhood cancer.

## Introduction

Wilms tumor (WT) is the most common renal malignancy of childhood. Black children of sub-Saharan African ancestry consistently show the highest incidence of WT worldwide at 11 cases per million (1). In sub-Saharan Africa, WT is reported as the second or third most common pediatric malignancy, which differs from its North American incidence (2). With the advent of cooperative trials, multimodal treatment regimens, and multidisciplinary care models, overall survival at 5-years for patients with WT in developed nations is now greater than 90% (3). However, alarming disparities in outcomes persist for children with WT residing in sub-Saharan African nations, with overall survival at 5-years as low as 25% (4). Over the past 50 years, basic descriptions of WT prevalence, treatment challenges, and poor outcomes for children living in resource-limited settings of sub-Saharan Africa have been published, with the principal focus in more recent years on social determinants of health as contributing factors to this profound cancer disparity (2, 5–7). Only in the last decade has examination of WT biology as a molecular determinant of health in these austere contexts begun to gain momentum (8–11). The principal purposes of this review were to provide a comprehensive summary of existing literature on WT in sub-Saharan Africans and to describe the current epidemiology, biologic features, treatment strategies, and outcomes in these at-risk and vulnerable children. Further, we aimed to highlight areas of study where additional clinical and molecular research are needed.

## Methods

Publications related to WT and sub-Saharan Africa were included in this review. Using PubMed, the search terms “Wilms tumor” and “Africa” retrieved 192 results. Publications were reviewed for relevance and content by both authors and were included if WT in sub-Saharan African nations or Black populations was described. Key findings and results were abstracted from each paper and summarized. Publications were categorized as (1): biologic or molecular determinants of health, if content included description of clinical and molecular or genomic features of WT in a sub-Saharan African or Black population, or (2) social determinants of health, if content included description of access to care, treatment abandonment, cultural beliefs, or healthcare infrastructure. Date of publication and country of origin were also recorded.

## Results

### Molecular Determinants of Health

Previous work has shown evidence of a biologic predisposition that may underlie an increased incidence of WT in children of Black sub-Saharan African descent (1). Specifically, a foundational study in 1984 showed that Black children living in the Greater Delaware Valley of the United States (i.e., Philadelphia, PA) were more likely to have congenital anomalies and syndromes associated with the development of WT. Specifically, a larger proportion of Black children had a WT-associated congenital anomaly, including aniridia, genito-urinary anomalies, Beckwith-Wiedemann Syndrome, and hemihypertrophy. Although not sequenced at the time of that seminal report, these developmental conditions associating with WT predisposition now have been attributed to alterations principally in two genes, *WT1* (11p13) and *WT2* (11p15.5) (12–14). Among younger patients, these authors reported a greater tendency for Black children to develop bilateral WT or to carry a tumor-associated anomaly. These features suggested a hereditary predisposition towards WT among Black children or less likely a greater susceptibility to toxins that induce germline mutations in these genes (15). After development and implementation of the National Wilms Tumor Study Group (NWTS) in 1969, which yielded 5 cooperative trials to optimize WT therapy, marked improvements in overall survival with reductions in treatment toxicity have since been realized (16–18). Moreover, a once significantly disparate survival gap for Black patients has now closed, at least in North America (3). However, Black populations globally continue to show greater frequencies to develop WT and to experience alarmingly poor survival in resource-constrained nations of sub-Saharan Africa. It was proposed in 1993 that, while global frequencies of WT were stable and not linked clearly or reproducibly with parental exposures to toxins, racial heredity and ancestry were greater determinants for development of WT than environmental exposures (1). To explore this concept of greater predisposition to develop WT among Black populations and potentially to harbor more treatment-resistant disease, both epidemiologic and somatic molecular differences between Black and White patients residing in Tennessee were explored. In Tennessee, Black children also appeared more susceptible than Whites to develop WT, and imaging mass spectrometry indeed identified peptide spectra from WT blastema and stroma that suggested race-specific molecular profiles (10).

Among sub-Saharan African populations, several initial studies described molecular features of WT that suggest a unique treatment-resistant and aggressive biology. These early studies aimed to quantify the frequency of p53 mutations that notoriously associate with diffuse anaplasia and more treatment-resistant disease. In one series of WT from Kenya, higher frequencies of p53 mutation were observed in comparison to White populations, and in accordance with previous literature, expression of p53 was associated with shorter survival period and unfavorable histology (19, 20). Additional molecular markers including E-cadherin, cadherin-11, alpha, beta and gamma-catenin were also studied within an African cohort. However, expression of these molecules did not show association with prognosis (21). Through multiple collaborations in Kenya and support from the Children’s Oncology Group, disparate molecular profiles were explored between North American and Kenyan WT specimens (8, 9, 11). An unbiased proteomic screen revealed unique protein signatures between North American Black, White, and Kenyan Wilms tumor specimens with excellent and race-specific clustering. Interestingly, peptide signatures from the North American WT specimens of Black and White patients appeared more similar than those between Black North American and Kenyan patients, which suggested a unique biologic composition within this latter sub-Saharan African population and likely greater genetic admixture in the former (11). Furthermore, sequencing of the top 10 winner peptides that associated with WT specimens from different race groups identified several interesting proteins and a novel association of Kenyan specimens with Fragile-X Related Protein – 1 (FXR1), which was subsequently characterized (22). FXR1 expression appeared to associate with undifferentiated cell types, specifically blastema, and may represent a pathway for cellular self-renewal hijacked from development (22). In Kenyan WT specimens, therefore, it is speculated that FXR1 emerged from the often blastemal-predominant cellular compartment in these cases that were analyzed commonly after neoadjuvant therapy and may represent a pathway for treatment resistance. Blastemal persistence after neoadjuvant therapy has been shown to be a poor prognostic feature, and indeed FXR1 has aligned with worse outcomes in several adult cancers (23).

Kenyan WT specimens have also been evaluated for histologic features and genomic alterations associated with somatic treatment resistance patterns. Specifically, Kenyan WT were analyzed for presence of diffuse anaplasia, which is an ominous harbinger of treatment resistance and failure, and in the majority of cases, is associated with alteration and mutation in *TP53*. While DAWT only comprises 5-8% of WT patients in high-income countries, anaplasia was present in 13% of Kenyan WT patients (9). Furthermore, an increased frequency of genetic and chromosomal alterations were uncovered in these specimens that have been associated with poor prognosis in high-income countries, including frequent mutations in p53, beta-catenin, and MYCN, loss of heterozygosity at 17p (which covers *TP53*) and 11q, and copy number gain at 1q (8, 9).

### Social Determinants of Health

Differences in access to care, cultural attitudes and beliefs, infrastructure, and health care delivery mechanisms only exacerbate the dismal outcomes for children having biologic features of treatment-resistant WT and residing in sub-Saharan Africa. Loss to follow up and treatment abandonment remain the most commonly reported social challenges that contribute to treatment failure across the continent (24, 25). Studies from multiple countries have aimed to implement multidisciplinary treatment models and standardized therapy to improve outcomes. Risk factors and challenges for providing optimal treatments have been described by treatment center and country (24, 25).

In the Collaborative Wilms Tumor Project, an adapted WT treatment guideline was implemented in multiple centers across sub-Saharan Africa, including the countries of Malawi, Cameroon, Ethiopia, Uganda and Ghana. The principal aim was to decrease abandonment of treatment and to improve outcomes (26). Using this multi-center regional collaborative network, program implementation was associated with significantly higher survival without evidence of disease at the end of treatment compared to baseline evaluations (68.5% vs. 52%) (26–28). Financial support for medical treatment was highlighted as a key strategy to decrease abandonment of treatment (28). In the first multicenter prospective study in sub-Saharan Africa, seven units participated from Senegal, Madagascar, Cameroon, Cote d’Ivoire, Mali, Togo, and Burkina Faso. After protocolized treatment of unilateral, localized, standard-risk WT, a three-year overall survival rate of 73% was observed (29). However, fifteen percent of the patients did not receive optimal treatment, and principal barriers included limited access to care. Specifically, decreased availability of pathology reports, decreased availability of chemotherapeutic drugs, and lack of access to radiotherapy were described (29).

In Kenya, we reported recently a 2-year event-free survival from WT as 52.7%, which rose from 35% from prior publications. However, loss to follow up in our series was 50%, which tempered enthusiasm (24). Other studies have reported similar rates of loss to follow-up at 42%. Also reported, late presentations of WT with advanced stages of disease contribute to decreased overall survival (30, 31). For those who completed standard therapy, however, 2-year event free survival has been documented as high as 94%, in accordance with overall survival in high-income nations. Insurance status and enrollment in the Kenyan National Hospital Insurance Fund (NHIF) was associated with lower hazard of death, which suggests the importance of health insurance (24). Risk factors for treatment abandonment in Kenya include financial constraints, lack of education about WT and necessity to complete treatment, and lack of drug availability (24, 25).

In Nigeria, clinical characteristics and outcomes have also been evaluated, showing larger than average tumor size at presentation in comparison to Caucasian children in high-income nations. A high mortality rate due to late clinical presentation, poor availability of chemotherapeutic agents, and inadequate follow up and treatment completion have been documented (32). Later studies evaluated outcomes following introduction of multidisciplinary team management and patient treatment stratification according to tumor histology. In this population, one third of patients were lost to follow up. Among patients who completed chemotherapy treatment, 5-year overall survival was 73.7%, but overall 5-year survival (abandonment-sensitive survival) remained low at 35.6% Barriers to care included public health measures that allowed early diagnosis, improvement of facilities, and adequate healthcare funding to receive standard therapy (33). Additional studies advocate for the need for additional health information and collaboration with institutions in high-income countries (34).

In Rwanda, nephroblastoma, or WT, was reported as the most common childhood cancer. Significant challenges to survival include unaffordable treatment, late presentation, and lack of trained staff and multidisciplinary collaboration. Recommendations for improvement again highlight improvement in patient education, free health care for children with cancer, international partnerships with tertiary care centers (35).

In Malawi, presentation at advanced stage and high recurrence rates are reported even with completion of therapy at 15% (36). An adapted WT treatment guideline and strategies to enable children to complete treatment were introduced. Two- and five-year event-free survivals remained decreased at 46 and 42%, respectively, in comparison to high income countries, and causes of treatment failure included abandonment of care for 7% of children, 15% with death during treatment, and 30% with disease-related deaths. Suggestions to optimize WT management in Malawi included strengthening social support programs, treatment compliance, nutrition, and modifications to reduce treatment-related deaths (37).

In South Africa, nutritional status was highlighted as a further prognostic feature impacting outcome from WT. Prevalence of malnutrition was as high as 66% using combined laboratory and anthropometric data. For this reason, early aggressive nutritional resuscitation for malnourished children in marginalized sub-Saharan African countries and populations was recommended (38). While presentation with advanced disease remained a challenge, treatment by multidisciplinary teams in Johannesburg showed improved survival outcomes relative to other sub-Saharan African nations (39). Furthermore, an additional study in South Africa showed that when treatment protocols employed in the United States were implemented in this African setting with robust surgical care, estimated 5-year overall survival was 94.4% (40).

The combined results of these publications from populations across sub-Saharan Africa highlight the need for improved access to care, availability of standard therapy for WT, supportive care, and patient education. These challenges remain significant and are cited as the primary determinant of decreased overall survival from WT in Africa in comparison to high-income nations (6, 7, 41, 42). Altogether, marginalized access to less than adequate therapies for malnourished children having advanced stage, treatment-resistant WT is exceedingly difficult to overcome, hence the horrific yet consistently poor survival in certain areas of sub-Saharan Africa.

### Timeline and Categorization of Publications

**Figure 1:**
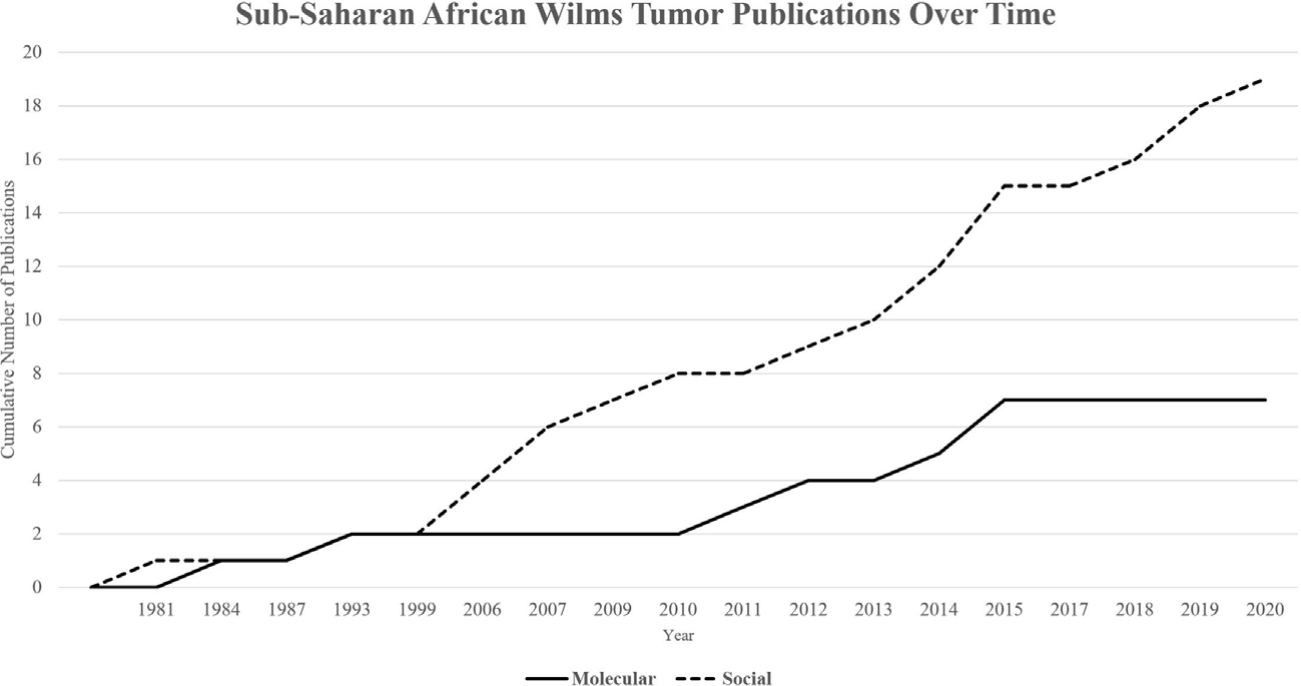
Cumulative publications over time by category. Publications on molecular determinants of health (Total = 7) have lagged in comparison to publications on social determinants of health (Total = 19) over time.

A total of 26 papers were included in this review. Since the first included publication in 1981, a total of 19 papers described social determinants of health and the impact of various financial, cultural, and structural barriers to optimal treatment in African populations on survival from WT (**Table 1**). Significant improvements have been made to address these barriers, including collaborative clinical trials, implementation of treatment protocols, multidisciplinary teams, international partnerships, and unique strategies for increasing access to care. Since the first publication in 1984, a total of 7 papers described molecular and genomic features within WT of sub-Saharan Africa (**Table 1**). A timeline underscores the lag to investigate molecular features for more optimal risk stratification and treatment assignment (**Figure 1**).

**Table 1:** Publications on Wilms tumor among sub-Saharan Africans.

Author	Journal/Date	Title	Country(ies)	Findings
Kyambi et al. (31)	EastAfrican Medical Journal/1981	The management of Wilms tumor in Kenya	Kenya	Late presentation and Loss to follow up (LTFU)
Kramer et al. (15)	Medical and Pediatric Oncology/1984	Racial Variation in incidence of Wilms tumor: relationship to congenital anomalies	United States	Genetic
Breslow et al. (1)	Medical and Pediatric Oncology/1993	Epidemiology of Wilms Tumor	Global	Genetic
Wessels et al. (38)	Pediatric Hematology Oncology/1999	Nutrition, morbidity, and survival in South African children with Wilms’ tumor	South Africa	Malnutrition
Ekenze et al. (34)	Annals of Oncology/2006	The challenge of nephroblastoma in a developing country	Nigeria	Health education and collaboration
Davidson et al. (40)	Pediatric Blood Cancer/2006	Wilms tumor experience in a South African Centre	South Africa	Treatment protocols and collaboration
Uba and Chirdan (32)	West African Journal of Medicine/2007	Wilms tumor: prognostic features in North Central Nigeria	Nigeria	LTFU
Rogers et al. (39)	European Journal of Pediatric Surgery/2007	Experience and outcomes of nephroblastoma in Johannesburg 1998-2003	South Africa	Late presentation, collaboration
Israels et al. (37)	Pediatric Blood Cancer/2009	Acute malnutrition is common in Malawian patients with Wilms tumor: a role for peanut butter	Malawi	Late presentation, LTFU
Wilde et al. (36)	African Journal of Paediatric Surgery/2010	Challenges and outcome of Wilms’ tumor management in a resource-constrained setting	Malawi	Malnutrition, late presentation, LTFU, drug availability
Axt et al. (10)	Journal of Surgical Research/2011	Race disparities in Wilms tumor incidence and biology	United States	Proteomic
Tenge et al. (30)	East African Medical Journal/2012	Management and outcome of patients with Wilms Tumor (nephroblastoma) at the MOI Teaching and Referral Hospital, Eldoret, Kenya	Kenya	Late presentation, LTFU, drug availability
Murphy et al. (9)	International Journal of Cancer/2012	Molecular characterization of Wilms’ tumor from a resource-constrained region of sub-Saharan Africa	Kenya	Proteomic, Histologic
Axt et al. (24)	Journal of Pediatric Surgery/2013	Wilms tumor survival in Kenya	Kenya	LTFU, cost of treatment
Israels et al. (41)	Pediatric Hematology Oncology/2014	Management of children with Wilms tumor in Africa and Europe; thoughts about costs, priorities and collaboration	The Netherlands and Malawi	LTFU, malnutrition, cost of treatment, collaboration
Israels et al. (41)	Pediatric Hematology Oncology/2014	Clinical trials to improve childhood cancer care and survival in sub-Saharan Africa	Sub-Saharan Africa	LTFU, cost of treatment, collaboration
Libes et al. (11)	Journal of the American College of Surgeons/2014	Race disparities in peptide profiles of North American and Kenyan Wilms Tumor Specimens	United States and Kenya	Proteomic
Libes et al. (25)	Pediatric Blood Cancer/2015	Risk factors for abandonment of Wilms tumor therapy in Kenya	Kenya	Cost of treatment, education, drug availability
Paintsil et al. (27)	European Journal of Cancer/2015	The Collaborative Wilms Tumor Africa Project; baseline evaluation of Wilms tumor treatment and outcome in eight institutes in sub-Saharan Africa	Malawi, Cameroon, Ghana, Ethiopia, Uganda	LTFU and death during treatment
Kanyamuhunga et al. (35)	Pan African Medical Journal/2015	Treating childhood cancer in Rwanda: the nephroblastoma example	Rwanda	Late presentation, cost of treatment, education, health care personnel
Atanda et al. (19)	African Journal of Paediatric Surgery/2015	Wilms tumor: determinants of prognosis in an African setting	Kenya	Histologic
Lovvorn et al. (8)	Genes, Chromosomes, and Cancer/2015	Genetic and chromosomal alterations in Kenyan Wilms Tumor	Kenya	Genomic
Israels et al. (26)	Pediatric Blood & Cancer/2018	Improved outcome at end of treatment in the Collaborative Wilms tumor Africa Project	Malawi, Cameroon, Ghana, Ethiopia, Uganda	Collaboration, LTFU, cost of treatment
Yao et al. (29)	Journal of Global Oncology/2019	Treatment of Wilms Tumor in Sub-Saharan Africa: Results of the Second French African pediatric Oncology Group Study	Senegal, Madagascar, Cameroon, Cote D’Ivoire, Mali, Togo, Burkina Faso	Treatment availability
Ekenze et al. (33)	Pediatric Blood & Cancer/2019	Continuing barriers to care of Wilms tumor in a low-income country	Nigeria	Late presentation, cost of treatment, health care facilities
Chagaluka et al. (28)	Pediatric Blood & Cancer/2020	Improvement of overall survival in the Collaborative Wilms Tumor Africa Project	Malawi, Cameroon, Ghani, Ethiopia, Uganda	Collaboration, LTFU, cost of treatment

## Discussion

WT disproportionately impacts Black children residing in sub-Saharan Africa and worldwide. This review illustrates significant progress in characterizing the clinical and molecular features of WT in sub-Saharan Africa and improving outcomes over the last 50 years, but clearly much work remains. Most sub-Saharan African nations categorized as low to middle income have seen improvement in survival outcomes since initial reports 4 decades ago, albeit not consistently near results from high income countries. Treatment abandonment remains a significant challenge reported by authors from multiple sub-Saharan African countries. The primary focus of research on WT in resource-limited regions of Africa is necessarily devoted to social determinants of health and decreasing barriers to care, of which there are many. Improving patient outcomes requires decreasing delayed presentation and diagnosis, increasing collaboration between interdisciplinary teams, improving access to pathology for treatment stratification, increasing availability of surgery, radiation, and chemotherapeutic agents, increasing adherence with follow up care, and comprehensive survivorship clinics. All of these factors are also likely impacted by finances, health literacy, and cultural beliefs. While these social determinants are certainly present in developed nations, it appears these inequities are exacerbated in low-resource settings of sub-Saharan Africa.

With the advent of targeted therapies, new frontiers of oncologic care focus on characterizing molecular signatures of disease with the goal of providing pathway- and cell-specific, personalized treatments. In high income nations, the focus of most WT research is optimizing therapy through further study of biomarkers associated with aggressive and treatment-resistance disease. This strategy to incorporate biologic features that assign risk of treatment failure within therapeutic regimens affords patients harboring a predictably sensitive WT to be exposed to less toxic therapy (4). The corollary of patients having a biologically high-risk WT will be assigned more appropriately intensive therapies. For example, specific genetic features of WT, including LOH for alleles spanning chromosomes 1p and 16q, are biomarkers that, when both present, associate with increased risk of relapse and death and have implications for more intensive management. Identifying additional prognostic biomarkers is an active area of study (43). Currently, the understanding of the genetic features of WT are based on specimens almost exclusively from patients in developed nations, which may not be generalizable to sub-Saharan African WT. Previous work has shown evidence of a predisposition among Black populations of sub-Saharan African ancestry to develop WT and that molecular markers associated with poor prognosis and treatment-resistant disease may well confound standard therapies. Further study and inclusion of African patients in molecular and genetic research is required to equitably advance treatment options for all patients with WT globally. Improved understanding of biologic features of WT in African populations will allow for risk stratification in parallel to the use of Children’s Oncology Group (COG) and International Society of Pediatric Oncology (SIOP) treatment protocols. Advancement of personalized therapies for WT in Africa will require collaborative efforts to characterize molecular features, determine prognostic significance, and evaluate the efficacy of tailoring chemotherapy intensity accordingly.

Limitations of this review include the incorporation of only published work and lack of sub-Saharan collaborators. The data reviewed may not reflect the entirety of research that has been conducted on Wilms Tumor in Africa, particularly studies that are ongoing or unpublished. Included publications were written by primarily sub-Saharan researchers and collaborators. However, the authors of this review do not practice in sub-Saharan Africa and therefore, may not capture additional perspectives or insights based on first-hand experience from within the region. Strengths of the review include the comprehensive summary of biologic and social factors relevant to understanding this pediatric health disparity, contemporary discussion of research trends over several decades, and suggestion of future directions to improve outcomes.

While social determinants are foundational and critical to improving outcomes for children with WT in sub-Saharan Africa, additional research is needed to better characterize disease at the genetic and molecular level. The results of this review show that publications on biologic and molecular features of disease in African WT are lagging in comparison to publications regarding social determinants of health. Sub-Saharan African children having WT are not only disproportionately impacted by structural and cultural barriers to care, but also may harbor a tumor biology that would benefit from additional risk stratification and personalized therapies. Indeed, even in high-income countries where access to appropriate care is assured, WT that acquire treatment-resistant molecular features are difficult enough to cure, let alone in resource-poor settings where social barriers abound, as described above. In order to address the persistent and widely reported health disparities in WT in Africa, efforts to address systems of care and decreasing treatment abandonment should remain a priority, in addition to improved understanding of Wilms tumorigenesis to advance personalized treatments.

## Author Contributions

HL contributed to the conception and design of the study. AA and HL contributed to the collection of the data, analysis, and manuscript development. All authors contributed to the article and approved the submitted version.

## Conflict of Interest

The authors declare that the research was conducted in the absence of any commercial or financial relationships that could be construed as a potential conflict of interest.
